# Draft Genome Sequence of “*Candidatus* Phytoplasma pruni” Strain CX, a Plant-Pathogenic Bacterium

**DOI:** 10.1128/genomeA.01117-15

**Published:** 2015-10-15

**Authors:** I.-M. Lee, J. Shao, K. D. Bottner-Parker, D. E. Gundersen-Rindal, Y. Zhao, R. E. Davis

**Affiliations:** a Molecular Plant Pathology Laboratory, U.S. Department of Agriculture, Beltsville, Maryland, USA; b Invasive Insect Biocontrol and Behavior Laboratory, U.S. Department of Agriculture, Beltsville, Maryland, USA

## Abstract

“*Candidatus* Phytoplasma pruni” strain CX, belonging to subgroup 16SrIII-A, is a plant-pathogenic bacterium causing economically important diseases in many fruit crops. Here, we report the draft genome sequence, which consists of 598,508 bases, with a G+C content of 27.21 mol%.

## GENOME ANNOUNCEMENT

Phytoplasmas are cell wall-less plant-pathogenic prokaryotes, which inhabit both insects (e.g., leafhoppers, planthoppers, and psyllids) and >1,000 plant species (1–3), causing numerous economically important diseases worldwide. In nature, insects serve as vectors that transmit phytoplasmas and spread diseases among plants. There are a vast number of diverse phytoplasma strains that are distributed on all continents. Phylogenetic analysis based on 16S rRNA gene sequences has indicated that phytoplasmas form a large discreet monophyletic clade paraphyletic to the genus *Acholeplasma* in the class *Mollicutes* (4). Because of the inability to readily cultivate phytoplasmas in cell-free medium, the provisional genus “*Candidatus* Phytoplasma” and also “*Candidatus* Phytoplasma spp.” were proposed to accommodate their classification (5). For finer classification of phytoplasmas, a scheme was proposed based on restriction fragment length polymorphism (RFLP) analysis of the 16S rRNA sequence that thus far includes 32 16S ribosomal (16Sr) groups and >200 subgroups (6, 7). Group 16SrIII represents one of the most diverse groups (8–10). “*Candidatus* Phytoplasma pruni” strain CX belongs to subgroup 16SrIII-A; strains in this subgroup cause severe disease of decline in many stone fruit trees (10). In order to understand the pathogenic nature of phytoplasmas, genomic sequencing has been the focus. Thus far, five phytoplasma genomes (belonging to 16SrI, 16SrX, and 16SrXII groups) have been fully sequenced (11–15). Draft genome sequences of four phytoplasmas belonging to 16SrIII-B (Italian clover phyllody, “*Candidatus* Phytoplasma” strain MA), 16SrIII-F (milkweed witches’ broom, “*Candidatus* Phytoplasma” strain MW1 and *Vaccinium* witches’ broom “*Candidatus* Phytoplasma” strain VAC), and 16SrIII-H (poinsettia branch-inducing “*Candidatus* Phytoplasma” strain JR1 = PoiBI), were published recently (16). Here, we report the draft genome sequence of “*Ca.* Phytoplasma pruni” strain CX.

“*Candidatus* Phytoplasma pruni” strain CX DNA was extracted from preparations of sieve cells isolated from infected periwinkle plants (*Catharanthus roseus*), as previously described (17), with the addition of RNase A digestion prior to the final phenol-chloroform extraction. Whole-genome paired-end sequencing was performed using the 454-GS Junior system (Roche Diagnostics, Indianapolis, IN). The original 177,537 reads were filtered using the BLAST(p/n). The reads were searched against custom BLAST databases containing “*Candidatus* Phytoplasma” sequences. This resulted in 132,205 reads that were assembled using the Newbler Assembler 2.9. The number of aligned reads was 130,704, and the number of aligned bases was 43,684,964. The average read coverage was 65×. The assembly resulted in 46 contigs >534 bp, with a total base value of 598,508 bases, which is in agreement with the sizes of other published group 16SrIII “*Candidatus* Phytoplasma” partial genomes (583 to 670 kb) (16, 18). The G+C content was 27.21 mol%. The *N*_50_ was 38,825 bases, and the largest contig was 93,855 bases. Use of the gene finder GeneMark.hmm on the largest 46 contigs resulted in the identification of 602 protein-coding genes.

The availability of the 16SrIII-A CX draft genome sequence combined with the other existing four draft genome sequences of group 16SrIII strains will facilitate the identification of specific genomic features of this group that may be responsible for the pathogeneses inflicted by various 16SrIII group “*Candidatus* Phytoplasma” strains. All five strains exhibit characteristic symptoms in their common host, *C. roseus.*

### Nucleotide sequence accession numbers.

This whole-genome shotgun project has been deposited in DDBJ/ENA/GenBank under accession no. LHCF00000000. The version described in this paper is the first version, LHCF01000000.
